# Association between perceived built environmental attributes and physical activity among adults in South Africa

**DOI:** 10.1186/s12889-017-4128-8

**Published:** 2017-02-20

**Authors:** Pasmore Malambo, Andre P. Kengne, Estelle V. Lambert, Anniza De Villers, Thandi Puoane

**Affiliations:** 1 Faculty of Community and Health Sciences School of Public Health, University of Western Cape, Cape Town, South Africa; 2 Non-communicable Diseases Unit, South African Medical Research Council, Cape Town, South Africa; 3 Division of Exercise Science and Sports Medicine, Department of Human Biology, Faculty of Health Sciences, University of Cape Town, Cape Town, South Africa

**Keywords:** Physical activity, Built environment, Transport, Leisure, Walking, South Africa

## Abstract

**Background:**

To investigate the association between perceived environmental attributes and leisure-time and transport-related physical activity.

**Methods:**

This was a cross-sectional survey involving 671 South Africans aged ≥35 years from urban and rural settings. International Physical Activity Questionnaire and Neighbourhood Walkability Scale were used to collect data. Multivariable logistic regressions were used to investigate the associations.

**Results:**

Significant urban vs. rural differences were apparent in the distribution of most attributes of neighborhood environment. After adjusting for gender, age, setting and relevant interaction terms, proximity to local stores was significantly associated with leisure-time physical activity (OR: 4.26; 95% CI, 1.00–18.08); while proximity to transit stops (2.44; 1.48–4.02), pleasant scenery (1.93; 1.07–3.46), sidewalks (2.36; 1.25–4.44), shade from trees (2.14; 1.19–3.85), traffic (2.17; 91.21–3.91) and well-lit streets (2.01; 1.04–3.89) were significantly associated with walking for leisure. Four-way intersections (4.54; 1.54–13.43), pleasant scenery (3.84; 1.35–10.99), traffic (0.28; 0.09–0.89), sidewalks (3.75; 1.06-13.27) and crosswalks were associated with transport related physical activity. Proximity to transit stops (2.12; 1.17–3.84) and well maintained sidewalks (2.69; 2.20–10.02) were significantly associated with total physical activity. Significant interactions by setting were apparent in some of the associations.

**Conclusion:**

Some, but not all attributes of a neighborhood environment were significantly associated in expected directions with the three physical activity domains in this mixed urban and rural population. This study highlights the need for policy strategies aimed at improving or maintaining these perceived environmental attributes to promote physical activity.

## Background

Regular physical activity (PA) is reported to be essential for the overall health and is associated with reduction in morbidity and mortality [1]. It is estimated that lack of physical activity accounts for between 3% and 4% of deaths among South Africans men and women respectively [2]. Consequently, 3.3% of all deaths in South Africa in 2000 were attributed to physical inactivity [3]. Moreover, 48% of South African men and 63% of African women were reported as being physically ‘inactive’ [4].

Walking for transportation can assist people in meeting recommended levels of physical activity [1]. Accordingly, residents living in highly walkable neighborhoods are more likely to walk for leisure than those living in low-walkable neighborhoods [5]. These findings are supported by evidence from others studies that showed aesthetic environment, convenience of facilities for walking, accessibility, and perception about traffic and busy roads to be associated with walking [6].

There is a growing body of international data showing that perceived built environments are associated with physical activity [7, 8] at a population level. Perceived built environment features such as proximity to destinations, sidewalks, the presence of physically active people in the neighborhood, higher residential density, neighborhood safety [7] and aesthetic quality [9] have been associated with moderate to vigorous physical activity and walking. Similarly, access to services, streets connectivity, pedestrian infrastructures, heavy traffic and a mix of utilitarian and recreational destinations have been linked to active travel, recreational physical activity [8] and leisure-time physical activity and leisure-time walking [9].

The design of built environment attributes that shapes and promotes active living is vital in modern society as this helps town planners and policy makers to make decisions that could potentially improve physical activity at the neighborhood level [10]. However, there remains a gap in the literature concerning the association between perceived built environment attributes and physical activity in an African context. For instance, with a few exceptions [11], most studies in this field originate from high income countries [12]. Therefore, the aim of this study was to investigate the association between perceived environmental attributes and leisure-time and transport-related physical activity in urban and rural communities in South Africa.

## Methods

### Study design

This was a cross-sectional analysis of the baseline data of the South African arm of the Prospective Urban and Rural Epidemiology (PURE) Study collected in 2009.

### Study population and setting

The study cohort included 2064 black South African men and women, aged 35–70 years, living in rural and urban sites, and was established in 2009. Communities selection purposefully favoured communities where a follow up of each respective cohort (urban vs rural) was feasible [13]. For the urban community (Langa in Cape Town), households were grouped into three development areas recognized administratively by the City of Cape Town Municipality. A street map obtained from the City of Cape Town was used to randomly select streets in each of these 3 areas. Once a street was selected, a systematic sampling of every second house was used to select potentially eligible participants for inclusion in the study. In the rural community (Mount Frere), the absence of delineated streets precluded following the same sampling approach used for the urban township. A cluster sample of houses in the community was therefore selected according to the division of areas determined by the clan heads. The inclusion criteria for both urban and rural were as follows: (1) households with a minimum of one member who was aged 35–70 years, (2) houses situated within an identified neighborhood and (3) houses without occupants with a disability that precluded them from walking. The sampling yielded 437 households in the urban community (1061 individuals) and 329 households in the rural community (1003 individuals). All households with eligible individuals were approached for recruitment, by trained field workers. For this study, all members in each household who met the criteria were used for analysis.

### Data collection

The PURE study used standardized, interviewer-administered questionnaires previously tested for anthropometric and biochemical measurements [13]. The study used the long version of the International Physical Activity Questionnaire (IPAQ) [14] and the Neighborhood Environment Walkability Scale (NEWS) questionnaire [15].

### Covariates

Socio-demographic information on age, sex, marital status, education level, and occupation were elicited from participants using a self-administered questionnaire. Participant’s age was grouped into 3 categories: 35– 44, 45–54, and 55 years or older. Marital status was classified as single, married, or divorced. Education level was classified as primary school education, secondary school education and tertiary school education. In this study, the occupation status was categorized as skilled (technicians, machine operators, clerks, skilled agriculture and fishery workers) and less skilled (homemaker, service, shop and market workers).

### Self-reported physical activity

The long version of the IPAQ was used to collect data on self-reported physical activity [16].

The IPAQ long form questionnaire assesses physical activity across a comprehensive set of domains including leisure time, domestic and gardening (yard), work-related, and transport-related physical activities, over the last 7 days. The IPAQ questionnaire was used to measure the frequency (days) and duration (in minutes) of vigorous-intensity PA, moderate-intensity PA, and walking-level PA separately. The total number of minutes per week in each PA category was computed (http://www.ipaq.ki.se). In the present study, four outcome variables were calculated: (1) leisure-time physical activity, (2) transport-related physical activity, (3) walking for leisure and (4) total physical activity. The four outcome variables were dichotomized into <150 min and ≥150 min according to WHO PA recommendations [17]. A 12-country, 14 - site study showed that the long IPAQ has excellent one-week test-retest reliability (pooled *r* = 0.81) and acceptable validity (pooled *r* = 0.33) when compared with accelerometer-measured physical activity [18].

### Self-perceived built environment

Participants completed interviewer-administered NEWS questionnaires [19], which assess the perceived built environment on the following selected variables: land use mix–access (4 items), walking/cycling infrastructure (3 items), aesthetics (3 items), traffic (3 items) and crime (3 items). Participants were instructed to consider neighborhood as the area within a 15–20 min walk from their home. These items used 4-point Likert scale-type of responses ranging from strongly disagree (1) to strongly agree (4). For the purpose of statistical analysis, a dichotomous variable was constructed. Responses to items were collapsed into categories: “disagree” (strongly disagree and somewhat disagree) and “agree” (somewhat agree and strongly agree). The NEWS questionnaire has been shown to be reliable and valid in reflecting neighbourhood walkability and the perceived neighbourhood environment, across a broad range of countries and settings [19].

#### Statistical analysis

The starting sample comprised 1016 participants of whom 345 were excluded for unacceptable levels of missing data [20]. Therefore, the final analytic sample comprised 671 participants. We used SPSS® version 22 for Windows (IBM Corp: Armonk New York) for all data analyses. Descriptive statistics were computed to measure frequencies for all categorical variables. In order to test for the association between perceived built environment and physical activity, univariable and multivariable models were constructed. In unadjusted logistic regression models, we tested the association between each perceived built environment item and the 4 physical activity outcomes. Potential confounders to be adjusted for in multivariable models, were first tested for their association with each of the outcome variables in univariable logistic regressions. These included age, sex, marital status, education and occupation. None of these variables were consistently associated with the 4 outcomes of interest (data not shown). Accordingly multivariable models were adjusted only for gender and age, under the assumption that confounding factor if any (both measured and unmeasured) would tend to be associated with either age or sex. In all regression models, all those who did not meet the 150 min per week recommended guidelines were used as reference category. In univariable models, the interactions between setting (urban vs. rural) and perceived built environment variables were tested by including in the same model the main effect of setting and built environment variable of interest, as well as their interaction term. Because of the many significant interactions, the interaction term of setting with each of the built environment variable was included in relevant multivariable models using the total sample. Furthermore, we have also presented the regression models stratified by setting. Statistical significance was set at *p* < 0.05.

## Results

Table 1 shows the descriptive characteristics of the sample. The sample included more women than men (76% vs. 24%) with no significance difference between urban and rural areas (*p* = 0.915). Over 34% of the subjects were aged 45–54 years, similarly in urban and rural areas (*p* = 0.303). In all, 41.3% of the participants were married with significant urban vs. rural difference (36.6% vs. 44.9%, *p* = 0.019). Over 54.4% were educated to a secondary school level and only 15.9% had skilled jobs and majority were black Africans (99.0%) from rural areas (56.8%), with no rural vs. urban differences in these characteristics (all p ≥0.130; Table 1).

**Table 1:** Descriptive characteristic of individuals by location

Variables (N (%))	Urban = 290	Rural = 381	*p*-value	All = 671
Covariates				
Sex			0.915	
Males	69 (23.8)	92 (24.1)		161 (24.0)
Females	221 (76.2)	289 (79.9)		510 (76.0)
Age			0.303	
35-44	87 (30.0)	134 (35.2)		221 (32.9)
45-54	100 (34.5)	129 (33.9)		229 (34.1)
55 +	103 (35.5)	118 (31.0)		221 (32.9)
Marital status			**0.019**	
Single	132 (45.5)	133 (34.9)		265 (39.5)
Married	106 (36.6)	171 (44.9)		277 (41.3)
Divorce	52 (17.9)	77 (20.2)		129 (19.2)
Education status			0.165	
Primary	86 (29.7)	190 (49.9)		276 (41.1)
Secondary	186 (64.1)	179 (47.0)		365 (54.4)
Tertiary	18 (6.2)	12 (3.1)		30 (4.5)
Occupation			0.558	
Less skilled	241 (83.1)	323 (84.8)		564 (84.1)
Skilled	49 (16.9)	58 (15.2)		107 (15.9)
Ethnicity			0.130	
Black African	285 (98.3)	379 (99.5)		664 (99.0)
Colored	5 (1.7)	2 (0.5)		7 (1.0)
Physical activity outcomes				
Leisure-time physical activity^a^			0.125	
< 150 min/week	181 (85.4)	194 (90.2)		375 (87.8)
≥ 150 min/week	31 (14.6)	21 (9.8)		52 (12.2)
Walking for leisure^a^			0.095	
< 150 min/week	153 (53.3)	226 (59.8)		379 (57.0)
≥ 150 min/week	134 (46.7)	152 (40.2)		286 (43.0)
Transport-related physical activity^a^			**0.018**	
< 150 min/week	199 (84.7)	255 (91.4)		454 (88.3)
≥ 150 min/week	36 (15.3)	24 (8.6)		60 (11.7)
Total physical activity			**<0.001**	
< 150 min/week	63 (21.7)	146 (38.3)		209 (31.1)
≥ 150 min/week	227 (78.3)	235 (61.7)		462 (68.9)

^a^sub sample less than 671 due to missing variables

Bold is significant *p* value

Only 12.2% of respondents met recommended physical activity guidelines (≥150 min/week) in the leisure-time domain. There was no difference in the prevalence of those persons accumulating at least 150 min/week of moderate-to-vigorous activity in leisure time, between urban and rural settings (14.6% vs 9.8%, *p* = 0.125). Overall, 57.0% of respondents did not accumulate at least 150 min per week of walking for leisure. This pattern was observed in both urban and rural settings (53.3% vs 57.8%), *p* = 0.095). For transport-related physical activity, the proportion of respondents achieving at least 150 min per week was 11.7% in the overall sample, and 15.3% in urban and 8.6% in rural areas, respectively (*p* = 0.018). Altogether, 68.9% of the respondents met the global recommendations of at least 150 min of moderate-to-vigorous physical activity per week (combining all domains), In fact, total moderate-to-vigorous activity prevalence was higher in the urban community (compared to the rural sample (78.3% vs.61.7%, *p* < 0.001; Table 1).

Table 2 illustrates the attributes of built environment overall and by location. The majority of respondents (68%) said they were able to do most of their shopping at a local store within walking distance from their homes. Destinations within neighborhoods were widely reported with more than 73% agreeing that there were many places to go within easy walking distance and 75% reporting that it was easy to walk to a transit stop from their residences. Approximately half the respondents (51%) felt that the distance between intersections was short, 54% agreed that there were many four-way intersections and 68% reported many alternative routes in their neighborhood. Despite over 54% agreeing that there were sidewalks on most streets, 52% reported sidewalks were not well maintained and not separated by grass from the streets. Almost half of the respondents indicated that there were no trees and a lack of pleasant scenery (interesting things) to see while walking and neighborhood was full of litter. Although 53% of the respondents reported a high volume of traffic along their streets, over 64% reported low volumes of traffic along nearby streets. Approximately half (51%) indicated that crosswalks did not help in crossing busy streets. The majority (57.1%) of the respondents reported that streets in their neighborhood were poorly lit at night, with 52% and 74% during the day/night respectively reporting that it was difficult to walk due to high crime rates (Table 2). In general, all built environment attributes were significantly different in urban and rural areas (all *p* < 0.001; Table 2).

**Table 2:** Descriptive characteristics of built environment attributes by location

Variables (N (%))	Urban = 290	Rural = 381	*p*-value	All = 671
Environmental attributes				
Land use mix-access				
I can do most of my shopping at local stores			**<0.001**	
Agree	243 (83.8)	218 (57.2)		461 (68.7)
Disagree	47 (16.2)	163 (42.8)		210 (31.3)
Stores are within easy walking distance of my home			**<0.001**	
Agree	243 (83.8)	218 (57.2)		461 (68.7)
Disagree	47 (16.2)	163 (42.8)		210 (31.3)
There are many places to go within easy walking distance of my home			**<0.001**	
Agree	258 (89.0)	237 (62.2)		495 (73.8)
Disagree	32 (11.0)	144 (37.8)		176 (26.2)
It is easy to walk to a transit stop (bus, train) from my home			**<0.001**	
Agree	258 (89.0)	249 (65.4)		507 (75.6)
Disagree	32 (11.0)	132 (34.6)		164 (24.4)
Street connectivity				
The distance between intersections in my neighborhood is usually short			**<0.001**	
Agree	238 (82.1)	87 (22.8)		325 (48.4)
Disagree	52 (17.9)	294 (77.2)		346 (51.6)
There are many four-way intersections in my neighborhood			**<0.001**	
Agree	240 (82.8)	125 (32.8)		365 (54.4)
Disagree	50 (17.2)	256 (67.2)		306 (45.6)
There are many alternative routes for getting from place to place in my neighborhood			**<0.001**	
Agree	248 (85.5)	208 (54.6)		456 (68.0)
Disagree	42 (14.5)	173 (45.4)		215 (32.0)
Places for walking and cycling				
There are sidewalks on most of the streets in my neighborhood			**<0.001**	
Agree	246 (84.8)	118 (31.0)		364 (54.2)
Disagree	44 (15.2)	263 (69.0)		307 (45.8)
The sidewalks in my neighborhood are well maintained			**<0.001**	
Agree	224 (77.2)	97 (25.5)		321 (47.8)
Disagree	66 (22.8)	284 (74.5)		350 (52.2)
There is a grass/dirt strip that separates the streets from the sidewalks in my neighborhood			**<0.001**	
Agree	199 (68.6)	122 (32.0)		321 (47.8)
Disagree	91 (31.4)	259 (68.0)		350 (52.2)
Neighborhood surroundings				
Trees give shade for the sidewalks in my neighborhood.			**<0.001**	
Agree	198 (68.3)	114 (29.9)		312 (46.6)
Disagree	92 (31.7)	267 (70.1)		359 (53.5)
There are many interesting things to look at while walking in my neighborhood.			**<0.001**	
Agree	210 (72.4)	104 (27.3)		314 (46.8)
Disagree	80 (27.6)	277 (72.7)		357 (53.2)
My neighborhood is generally free from litter.			**<0.001**	
Agree	173 (59.7)	107 (28.1)		280 (41.7)
Disagree	117 (40.3)	274 (71.9)		391 (58.3)
Safety from traffic				
There is so much traffic along the street I live such that it makes it difficult to walk in my neighborhood			**<0.001**	
Agree	235 (81.0)	127 (33.3)		362 (53.9)
Disagree	55 (19.0)	254 (66.7)		309 (46.1)
There is so much traffic along nearby streets that it makes it difficult to walk in my neighborhood			**<0.001**	
Agree	179 (61.7)	61 (16.0)		240 (35.8)
Disagree	111 (38.3)	320 (84.0)		431 (64.2)
The crosswalks in my neighborhood help walkers feel safe crossing busy streets			**<0.001**	
Agree	228 (78.6)	98 (25.7)		326 (48.6)
Disagree	62 (21.4)	283 (74.3)		345 (51.4)
Safety from crime				
My neighborhood streets are well lit at night			**<0.001**	
Agree	234 (80.7)	54 (14.2)		288 (42.9)
Disagree	56 (19.3)	327 (85.8)		383 (57.1)
The crime rate in my neighborhood makes it unsafe to go on walks during the day			**<0.001**	
Agree	22 (76.2)	133 (34.9)		354 (52.8)
Disagree	69 (23.8)	248 (65.1)		317 (47.2)
The crime rate in my neighborhood makes it unsafe to go on walks at night			**<0.001**	
Agree	243 (83.8)	257 (67.5)		500 (74.5)
Disagree	47 (16.2)	124 (32.5)		171 (25.5)

Bold is significant *p* value

The univariable regression analyses in the overall sample are summarized in Table 3, showing some significant associations between built environment attributes and physical activity, but also a number of significant interactions, by setting, for those associations. In unadjusted regression analyses stratified by setting (Table 4), among urban dwellers who agreed that there were many four-way intersections, sidewalks were well maintained and separated from streets by grass and clean neighborhood were positively associated with leisure-time physical activity (all *p* < 0.05), Table 4. In addition, transit stop, four-way intersections, all infrastructure variables, pleasant scenery and well-lit streets at night were positively associated with walking for leisure (all *p* < 0.05). This pattern was almost similar for total physical activity (Table 4). Meanwhile among rural counterparts, shade from trees and pleasant scenery were positively associated with leisure-time physical activity (both *p* < 0.05). In addition, alternative routes and crosswalks were both associated with walking for leisure (*p* < 0.05). Those who agreed that streets were well maintained and separated from street by grass were more likely to participate in transport related physical activity (both *p* < 0.05), Table 4. Similarly, alternative routes in rural area were positively associated with total physical activity. Conversely, among urban respondents, high traffic volume and crime rate at night were inversely associated with walking for leisure (both *p* < 0.05. Meanwhile in the rural area, high crime rate at night and traffic volume were negatively associated with leisure-time physical activity, walking for leisure and total physical activity (all *p* < 0.05; Table 4), respectively.

**Table 3:** Odd ratios and 95% confidence intervals from crude logistic regressions between environmental factors and physical activity in the overall sample

					Setting * built environment factors			
Variables	LTPA	WL	TRPA	TPA	Setting*LTPA p	Setting *WL p	Setting *TRPA p	Setting *TPA p
Built environmental factors (agree vs disagree)								
Land use mix-access								
I can do most of my shopping at local stores	1.70 (0.82-3.51)	0.86 (0.61-1.22)	0.85 (0.45-1.50)	1.05 (0.74-1.49)	0.053	0.807	0.755	0.942
Stores are within easy walking distance of my home	1.70 (0.82-3.51)	0.86 (0.61-1.22)	0.85 (0.45-1.50)	1.05 (0.74-1.49)	0.053	0.807	0.755	0.942
There are many places to go within easy walking distance of my home	1.21 (0.58-2.52)	0.97 (0.68-1.40)	0.96 (0.52-1.76)	1.12 (0.78-1.62)	0.536	0.736	0.891	0.149
It is easy to walk to a transit stop (bus, train) from my home	2.20* (1.01-8.30)	1.71** (1.17-2.50)	2.36* (1.09-5.12)	2.67*** (1.57-3.26)	0.478	0.052	0.850	0.781
Street connectivity								
The distance between intersections in my neighborhood is usually short	1.33 (0.73-2.40)	1.23 (0.90-1.69)	2.05* (1.17-3.59)	1.72** (1.23-2.4)	0.056	0.824	0.935	0.742
There are many four-way intersections in my neighborhood	1.10 (0.60-1.10)	0.75 (0.54-1.03)	0.84 (0.49-1.44)	1.31 (0.95-1.82)	**0.018**	0.185	0.421	**0.007**
There are many alternative routes in my neighborhood	1.91 (0.90-4.05)	0.75 (0.54-1.06)	1.11(0.61-2.01)	1.07 (0.75-1.51)	0.979	0.066	0.730	**0.015**
Infrastructure for walking and cycling								
There are sidewalks on most of the streets in my neighborhood	1.31 (0.72-2.30)	1.54** (1.12-2.12)	1.11 (0.65-1.92)	1.10 (0.79-1.53)	0.063	**0.002**	0.847	**0.015**
The sidewalks in my neighborhood are well maintained	1.31 (0.72-2.38)	0.76 (0.55-1.04)	0.98 (0.57-1.68)	0.78 (0.56-1.08)	**0.004**	**<0.001**	**0.036**	**0.034**
There is a grass that separates the streets from the sidewalks	1.56 (0.86-2.82)	1.40* (1.01-1.92)	0.82 (0.48-1.41)	1.09 (0.78-1.51)	**0.016**	**0.001**	0.134	**<0.001**
Aesthetics								
Trees give shade for the sidewalks in my neighborhood.	1.39 (0.77-2.49)	0.96 (0.69-1.31)	1.43 (0.83-2.46)	1.33 (0.96-1.85)	**0.026**	0.614	0.551	0.963
There are many interesting things to look at while walking	0.89 (0.50-1.60)	0.83 (0.61-1.15)	1.90* (1.09-3.32)	1.43* (1.03-2.00)	**0.020**	0.214	0.412	0.824
My neighborhood is generally free from litter.	0.94 (0.52-1.67)	1.49* (1.08-2.06)	1.61 (0.94-2.76)	**1.38** (0.99-1.94)	**0.034**	0.963	0.105	0.098
Safety from traffic								
Too much traffic along the street I live in makes it difficult walk	0.66 (0.37-1.78)	0.48*** (0.35-0.66)	1.11 (0.64-1.92)	0.98 (0.71-1.36)	0.938	0.788	0.919	0.148
Too much traffic along nearby streets makes it difficult walk	0.77 (0.42-1.40)	0.74 (0.53-1.04)	1.10 (0.64-1.91)	0.81 (0.58-1.45)	0.180	0.726	0.762	0.497
The crosswalks help walkers feel safe crossing busy streets	1.14 (0.63-2.05)	0.81(0.59-1.11)	0.86 (0.50-1.47)	1.31 (0.94-1.81)	0.670	0.668	0.852	0.640
Safety from crime								
My neighborhood streets are well lit at night	1.37 (0.76-2.46)	1.75 (0.91-3.36)	1.78* (1.04-3.07)	2.07*** (1.46-2.92)	0.925	**0.015**	0.299	0.165
The crime rate makes it unsafe to go on walks during the day	0.75 (0.42-1.35)	1.30 (0.68-2.49)	0.75 (0.44-1.31)	0.75 (0.54-1.04)	**0.015**	0.391	0.248	0.596
The crime rate makes it unsafe to go on walks at night.	0.78 (0.37-1.61)	0.41* (0.18-0.90)	0.60 (0.29-1.22)	0.89 (0.61-1.30)	0.967	0.308	0.678	0.086

*LTPA* Leisure time physical activity, *WL* Walking for leisure, *TRPA* Transport related physical activity, *TPA* Total physical activity, *P p*-value; * *p* < 0.05; ** *p* < 0.01; *** *p* < 0.001

Bold is significant *p* value

**Table 4:** Odd ratios and 95% confidence intervals from crude logistic regression between environmental factors and physical activity (ref <150mins/week) in urban and rural participants

Variables	Urban				Rural			
	LTPA	WL	TRPA	TPA	LTPA	WL	TRPA	TPA
Environmental factors (agree vs disagree)								
Land use mix-access								
I can do most of my shopping at local stores	**7.19** (0.95-54.55)	1.37 (0.73-2.56)	1.62 (0.70-3.75	1.43 (0.63-3.23)	0.80 (0.32-2.00)	1.24 (0.82-1.88)	1.34 (0.58-3.10)	1.34 (0.88-2.05)
Stores are within easy walking distance of my home	**7.19** (0.95-54.55)	1.37 (0.73-2.56)	1.62 (0.70-3.75	1.43 (0.63-3.23)	0.80 (0.32-2.00)	1.24 (0.82-1.88)	1.34 (0.58-3.10)	1.34 (0.88-2.05
There are many places to go within easy walking distance of my home	1.50 (0.33-6.86)	1.34 (0.64-2.79)	1.30 (0.46-3.68)	0.68 (0.30-1.55)	0.86 (0.34-2.17)	1.16 (0.76-1.76)	1.43 (0.61-3.31)	1.34 (0.87-2.07)
It is easy to walk to a transit stop (bus, train) from my home	1.70 (0.38-7.70)	2.03**(1.19-2.81)	2.22 (0.50-9.87)	1.83** (1.19-2.81)	3.66 (0.83-16.27)	0.86 (0.41-1.80)	1.87 (0.72-4.88)	**2.08** (0.94-4.54)
Street connectivity								
The distance between intersections in my neighborhood is usually short	2.88 (0.65-12.73)	1.03 (0.56-1.88)	1.71 (0.57-5.14)	1.25 (0.62-2.53)	0.43 (0.12-1.53)	1.12 (0.69-1.83)	1.61 (0.63-4.08)	1.09 (0.66-1.79)
There are many four-way intersections in my neighborhood	3.70* (1.05-12.99)	2.17**(1.37-3.45)	1.61 (0.67-3.87)	1.57* (1.02-2.43)	0.21 (0.03-1.59)	1.29 (0.70-2.38)	2.87 (0.95-8.65)	**0.52** (0.26-1.02)
There are many alternative routes in my neighborhood	1.70 (0.38-7.70)	0.92 (0.47-1.81)	1.03 (0.37-2.87)	0.56 (0.27-1.16)	1.66 (0.64-4.30)	1.94**(1.28-2.94)	1.30 (0.56-3.00)	1.59* (1.05-2.43)
Infrastructure for walking and cycling								
There are sidewalks on most of the streets in my neighborhood	4.15 (0.54-31.98)	3.55***(2.15-5.88)	1.68 (0.70-4.03)	2.02** (1.30-3.15)	0.42 (0.12-1.49)	0.99 (0.52-1.90)	1.90 (0.73-4.96)	0.70 (0.34-1.45)
The sidewalks in my neighborhood are well maintained	10.52* (1.40-79.31)	3.41***(1.98-5.88)	0.83 (0.36-1.94)	3.31***(2.06-5.34)	0.26 (0.06-1.15)	0.76 (0.44-1.34)	5.12* (1.18-22.30)	1.33(0.66-2.67)
There is a grass that separates the streets from the sidewalks	2.78* (1.02-7.58)	2.63***(1.64-4.23)	1.11 (0.52-2.35)	2.51***(2.06-5.34)	0.44 (0.14-1.35)	0.82 (0.50-1.36)	3.07* (1.02-9.25)	0.62(0.35-1.11)
Aesthetics								
Trees give shade for the sidewalks in my neighborhood.	0.70 (0.30-1.66)	1.22 (0.74-2.00)	1.35 (0.62-2.96)	1.00(0.55-1.82)	3.95* (1.13-13.86)	1.02 (0.65-1.60)	0.93 (0.37-2.34)	0.98(0.63-1.54)
There are many interesting things to look at while walking	0.65 (0.25-1.67)	1.75* (1.09-2.83)	2.06 (0.81-5.20)	1.04(0.56-1.95)	5.24* (1.19-23.18)	1.12 (0.67-1.88)	1.20 (0.49-2.92)	0.95(0.60-1.52)
My neighborhood is generally free from litter.	**2.33** (0.99-5.48)	1.32 (0.82-2.12)	2.04 (0.95-4.36)	0.75(0.42-1.33)	0.50 (0.16-1.55)	1.34 (0.85-2.11)	0.71 (0.26-1.97)	1.40(0.88-2.24)
Safety from traffic								
Too much traffic along the street I live in makes it difficult walk	0.39* (0.16-0.95)	0.39**(0.21-0.71)	0.83 (0.35-1.96)	1.01(0.49-2.05)	0.42 (0.14-1.29)	0.35***(0.22-0.56)	0.78 (0.32-1.88)	054**(0.35-.84)
Too much traffic along nearby streets makes it difficult walk	0.76 (0.35-1.65)	0.66 (0.41-1.06)	0.74 (0.36-1.52)	0.91(0.51-1.62)	0.17 (0.22-1.31)	0.57 (0.32-1.04)	0.91 (0.30-2.79)	0.69(0.40-1.20)
The crosswalks help walkers feel safe crossing busy streets	1.26 (0.47-3.34)	1.40 (0.80-2.46)	1.86 (0.84-4.12	1.06(0.53-2.11)	1.12 (0.41-3.03)	1.65* (1.01-2.68)	2.12 (0.70-6.42)	1.29(0.81-2.06)
Safety from crime								
My neighborhood streets are well lit at night	1.01 (0.36-2.84)	2.07* (1.16-3.70)	0.95 (0.35-2.33)	1.99*(1.04-3.80)	0.94 (0.30-2.95)	0.74 (0.41-1.34)	1.97 (0.69-5.68)	1.07(0.59-1.93)
The crime rate makes it unsafe to go on walks during the day	1.34 (0.52-3.47)	0.67 (0.39-1.15)	0.72 (0.32-1.60)	1.12(0.59-2.13)	0.19**(0.05-0.66)	0.90 (0.59-1.39)	**1.43 (0.61-3.35)**	0.91(0.59-1.40)
The crime rate makes it unsafe to go on walks at night.	0.85 (0.27-2.61)	0.48* (0.25-0.92)	0.78 (0.28-2.16)	1.29(0.63-2.66)	0.87 (0.32-2.36)	0.72 (0.47-1.12)	0.56 (0.21-1.60)	0.61*(0.39-.96)

*LTPA* Leisure time physical activity, *WL* Walking for leisure, *TRPA* Transport related physical activity, *TPA* Total physical activity; * *p* < 0.05; ** *p* < 0.01; *** *p* < 0.001

Bold is significant *p* value

The gender, age and site (and relevant interact terms in the overall sample) adjusted models are shown in Table 5 and 6. In these models applied to overall sample, significant associations were apparent between proximity to local stores and leisure time physical activity (4.26; 1.00-18.08), proximity to transit stop and walking for leisure (2.44; 1.48-4.02), proximity to transit stop and total physical activity (2.12; 1.17-3.84), availability of four-way intersections and transport related physical activity (4.54; 1.54-13.43), interesting things and walking for leisure (1.93; 1.07-3.46), interesting things and transport related physical activity (3.84; 1.35-10.93), and too much traffic along the street and leisure time related physical activity (0.28; 0.09-0.89). These associations were found in both urban and rural areas, although not always of the same magnitude, and not always statistically significant in each setting, separately (Table 6).

**Table 5:** Odd ratios and 95% confidence intervals from adjusted logistic regression between environmental factors and physical activity in urban and rural participants in the overall sample

	Physical activity outcomes			
Variables^a^	LTPA	WL	TRPA	TPA
Environmental factors (agree vs disagree)				
Land use mix-access				
I can do most of my shopping at local stores	4.26* (1.00-18.06)	1.38 (0.76-2.51)	0.87 (0.35-2.16)	0.73(0.39-1.38)
There are many places to go within easy walking distance of my home	0.46 (0.14-1.84)	1.01 (0.54-1.90)	0.81 (0.27-2.46)	0.93 (0.46-1.87)
It is easy to walk to a transit stop (bus, train) from my home	3.72 (0.85-16.28)	2.44*** (1.48-4.02)	2.58 (0.77-.8.66)	2.12*(1.17-3.84)
Street connectivity				
The distance between intersections in my neighborhood is usually short	1.01 (0.27-3.80)	1.45 (0.75-2.80)	**3.53** (0.96-12.91)	0.68 (0.34-1.38)
There are many four-way intersections in my neighborhood	0.85 (0.22-3.21)	0.59 (0.31-1.14)	4.54** (1.54-13.43)	1.85 (0.93-3.65)
There are many alternative routes in my neighborhood	2.37 (0.63-8.92)	0.82 (0.48-1.40)	1.35 (0.49-3.74)	0.95 (0.51-1.76)
Infrastructure for walking and cycling				
There are sidewalks on most of the streets in my neighborhood	0.71 (0.17-2.89)	2.36** (1.25-4.44)	3.75* (1.06-13.27)	1.13 (0.56-2.27)
The sidewalks in my neighborhood are well maintained	0.52 (0.15-1.81)	1.12 (0.55-2.27)	0.58 (0.19-1.73)	4.69*** (2.20-10.02)
There is a grass that separates the streets from the sidewalks	1.85 (0.71-4.85)	0.83 (0.45-1.54)	0.96 (0.42-2.23)	1.30 (0.74-2.28)
Aesthetics				
Trees give shade for the sidewalks in my neighborhood.	1.07 (0.37-3.05)	2.14* (1.19-3.85)	1.02 (0.38-2.78)	1.11 (0.59-2.09)
There are many interesting things to look at while walking	0.72 (0.24-2.22)	1.93* (1.07-3.46)	3.84* (1.35-10.93)	0.92 (0.49-1.73)
My neighborhood is generally free from litter.	1.50 (0.65-3.45)	1.34 (0.85-2.11)	1.56 (0.75-3.23)	1.04 (0.64-1.68)
Safety from traffic				
Too much traffic along the street I live in makes it difficult walk	0.28* (0.09-0.89)	2.17* (1.21-3.91)	0.76 (0.28-2.06)	0.93 (0.50-1.74)
Too much traffic along nearby streets makes it difficult walk	1.28 (0.46-3.58)	1.05 (0.54-2.03)	0.89 (0.36-2.23)	0.90 (0.49-1.66)
The crosswalks help walkers feel safe crossing busy streets	0.64 (0.20-1.98)	0.73 (0.38-1.43)	4.11** (1.47-11.50)	1.08 (0.56-2.07)
Safety from crime				
My neighborhood streets are well lit at night	0.70 (0.23-2.18)	2.01* (1.04-3.89)	1.52 (0.43-5.38)	2.50 (1.24-5.04)
The crime rate makes it unsafe to go on walks during the day	0.44 (0.17-1.17)	0.87 (0.49-1.52)	0.89 (0.34-2.36)	1.10 (0.61-1.99)
The crime rate makes it unsafe to go on walks at night.	1.42 (0.46-4.41)	0.91 (0.54-1.51)	1.70 (0.57-5.04)	0.83 (0.46-1.52

*LTPA* Leisure time physical activity, *WL* Walking for leisure, *TRPA* Transport related physical activity, *TPA* Total physical activity;^a^ adjusted for age, sex, site and the interaction term of site with each of the predictors of interest; * *p* < 0.05; ** *p* < 0.01; *** *p* < 0.001

Bold is significant *p* value

**Table 6:** Odd ratios and 95% confidence intervals from adjusted logistic regression between environmental factors and physical activity in urban and rural participants (ref <150mins/week)

	Urban				Rural			
Variables	LTPA	WL	TRPA	TPA	LTPA	WL	TRPA	TPA
Environmental factors (agree vs disagree)								
Land use mix-access								
I can do most of my shopping at local stores	6.76 (0.68-66.10)	1.20 (0.35-4.07)	0.38 (0.11-1.26)	0.60 (0.15-2.41	4.18 (0.40-44.05)	1.81 (0.64-5.18	0.86 (0.16-4.73)	2.14 (0.69-6.65)
There are many places to go within easy walking distance of my home	0.33 (0.03-3.46)	0.51 (0.07-3.73)	1.52 (0.23-9.97)	1.75 (0.20-15.43)	0.57 (0.08-3.98)	0.73 (0.26-2.04)	0.25 (0.05-1.26)	0.57 (0.18-1.78)
It is easy to walk to a transit stop (bus, train) from my home	1.21 (0.15-9.72)	4.80***(2.08-11.12)	1.41 (0.18-11.14)	4.12 (0.52-32.79)	9.78* (1.05-91.26)	1.80 (0.30-10.77)	2.12 (0.60-7.49)	3.84** (1.75-8.46)
Street connectivity								
The distance between intersections in my neighborhood is usually short	1.86 (0.23-14.92)	2.21 (0.60-8.10)	8.10* (1.12-58.79)	1.47 (0.23-9.25)	2.40 (0.23-25.40)	0.42 (0.11-1.59)	3.61 (0.57-22.92)	1.02 (0.32-3.27)
There are many four-way intersections in my neighborhood	5.61 (0.42-9.02)	0.88 (0.21-3.68)	6.62*(1.42-30.82)	16.57* (1.78-154.49)	0.13 (0.01-2.25)	1.27 (0.40-4.09)	2.68 (0.41-17.39)	2.17 (0.65-7.29)
There are many alternative routes in my neighborhood	1.02 (0.12-9.02)	0.76 (0.16-3.57)	1.17 (0.24-5.75)	0.34 (0.05-2.39)	3.39 (0.65-16.62)	0.75 (0.30-1.86)	**3.57** (0.95-13.38)	1.10 (0.43-2.82)
Infrastructure for walking and cycling								
There are sidewalks on most of the streets in my neighborhood	2.19 (0.16-31.09)	3.36* (1.07-10.51)	0.25 (0.04-1.59)	0.21 (0.03-1.72)	1.26 (0.09-17.54)	0.44 (0.09-2.21)	1.36 (0.24-7.66)	0.49 (0.19-1.25)
The sidewalks in my neighborhood are well maintained	8.57 (0.56-30.08)	8.06* (1.58-41.09)	0.33 (0.07-1.64)	**4.49** (0.98-20.62)	0.07 (0.00-6.66)	0.67 (0.22-2.08)	7.49 (0.36-154.60)	0.92 (0.22-3.77)
There is a grass that separates the streets from the sidewalks	2.27 (0.63-8.21)	1.53 (0.59-3.97)	0.64 (0.22-1.83)	2.16 (0.75-6.23)	4.16 (0.14-23.44	1.40 (0.39-5.04)	0.70 (0.07-7.02)	0.37 (0.08-1.60)
Aesthetics								
Trees give shade for the sidewalks in my neighborhood.	0.84 (0.20-3.61)	0.96 (0.33-2.78)	0.47 (0.12-1.91)	0.96 (0.16-5.91)	0.11 (0.01-1.49)	**2.94** (0.10-8.71)	1.53 (0.29-7.10)	**3.05** (0.97-9.60)
There are many interesting things to look at while walking	1.14 (0.24-5.51)	3.38* (1.25-9.15)	12.49** (2.04-76.43)	0.85 (0.14-5.17)	0.63 (0.05-8.83)	30.74 (0.24-2.29)	3.97 (0.81-19.50)	0.76 (0.22-2.63)
My neighborhood is generally free from litter.	1.88 (0.56-6.30)	1.15 (0.49-2.69)	2.63 (0.88-7.87)	4.01* (1.23-13.03)	0.40 (0.05-3.02)	1.71 (0.72-4.03)	0.29 (0.05-1.67)	0.70 (0.28-1.75)
Safety from traffic								
Too much traffic along the street I live in makes it difficult walk	0.19* (0.04-0.80)	0.39 (0.12-1.29)	0.81 (0.22-2.91)	0.96 (0.20-4.66)	0.19 (0.02-2.18)	1.07 (0.37-3.09)	1.39 (0.29-7.34)	1.26 (0.42-3.81)
Too much traffic along nearby streets makes it difficult walk	1.18 (0.31-4.41)	0.59 (0.20-1.73)	0.65 (0.21 (2.02)	0.52 (0.15-1.84)	0.44 (0.01-15.01)	1.22 (0.31-4.73)	0.51 (0.04-5.93)	0.68 (0.19-2.42)
The crosswalks help walkers feel safe crossing busy streets	0.65 (0.13-3.12)	0.59 (0.19-1.85)	2.97 (0.77-11.48)	0.19 (0.03-1.14)	**24.41**(0.75-94.15)	0.76 (0.22-2.60)	14.34* (1.23-67.56)	0.95 (0.27-3.38)
Safety from crime								
My neighborhood streets are well lit at night	0.40 (0.08-1.99)	10.26** (1.92-54.80)	0.72 (0.12-4.31)	8.58** (1.72-42.81)	0.91 (0.11-7.62)	0.66 (0.21-1.90)	5.61 (0.64-49.36)	2.31 (0.61-8.74)
The crime rate makes it unsafe to go on walks during the day	0.82 (0.19-3.55)	0.63 (0.21-2.49)	0.39 (0.10-1.49)	1.79 (0.50-6.40)	0.04*(00.0-0.65)	1.08 (0.41-2.85)	2.07 (0.35-12.38)	0.64 (0.25-1.68)
The crime rate makes it unsafe to go on walks at night.	0.40 (0.07-2.46)	0.63 (0.16-2.49)	2.08 (0.40-10.89)	0.51 (0.10-2.59)	2.17 (0.39-11.96)	0.69 (0.29-1.66)	2.53 (0.58-11.16)	0.84 (0.34-2.04)

*LTPA* Leisure time physical activity, *WL* Walking for leisure, *TRPA* Transport related physical activity, *TPA* Total physical activity; ^a^adjusted for age and sex; *p < 0.05; **p < 0.01; ***p < 0.001; bold, on borderline

In the overall sample significant associations were also found between availability of sidewalks and walking for leisure (2.36; 1.25-4.44), availability of sidewalks and transport related physical activity (3.75; 1.06-13.27), availability of maintained sidewalks and total physical activity (4.69; 2.20-10.02), shaded (trees) sidewalks and walking for leisure (2.14; 1.19-3.85), too much traffic along the street and walking for leisure (2.17; 91.21-3.91), crosswalks and transport related physical activity (4.11; 1.47-11.50), and well lighted streets at night and walking for leisure (2.01; 1.04-3.89). When rural and urban settings were considered separately, these associations were not always in the same direction, not always significant, nor did that always result in significant interactions by setting (Table 6).

Finally, some significant associations were found in setting specific analyses, but not in the overall sample. These included the associations of leisure time physical activity with transit stops and crime rates in rural setting, the association of walking for leisure with availability of well-maintained sidewalks in urban setting, the associations of total physical activity with availability of four-way intersections, neighborhoods free from litter and well-lit streets at night in urban settings, and shaded sidewalks in rural setting (Table 6).

## Discussion

A proportion of subjects reached 150-min per week threshold in total physical activity outcomes. After adjusting for gender, age and site (including interaction terms), attributes of the built environment including proximity to local stores, transit stops, four-way intersections, the availability of sidewalks and crosswalks, shade from trees and pleasant scenery, as well as a high volume of traffic, well-lit streets at night and concerns of personal safety during the day were associated with meeting physical activity guidelines of accumulating at least 150 min of moderate-to-vigorous activity per week, among the urban and rural South Africans surveyed.

This study supports the growing evidence that proximity and ease of access to destinations and services such as local stores and transit stops from residences are linked to more active living including [21] leisure-time physical activity, walking for leisure and total physical activity. These results are aligned with the results from an 11-country, International Physical Activity and Environment Network study [22]. Similarly, a study in China found access to physical activity destinations were related to leisure-time physical activity [9]. Access to services has been associated with sufficient walking in some studies [23] but not all [24]. Although the current study did not ask the participants about ownership of cars, it is unlikely that many people owned one, and thus walking for transport is their only means of travel [25].

We found that the occurrence of short distances between intersections and 4-way intersections in the neighborhood was significantly associated with respondents achieving 150 min or more of transport-related physical activity. This mirrors outcomes in most existing studies [6], with one exception [26]. The latter study, however, was confined to the university environment and consequently their perception of street connectivity may have been different from other studies [26]. Nevertheless, similar to other studies, the possible interpretation for a positive association would be that the availability of well-connected streets provides direct routes and safety for commuters, which ultimately increases the opportunity to walk. In a South African context, and particularly in urban areas where most of the streets are tarred and well connected, it facilitates residents’ use of streets for transport related physical activity.

Similar to existing studies [27], we also found that the presence of sidewalks on most streets was positively associated with walking for leisure. Likewise, better-quality sidewalks have been associated with both walking and meeting physical activity recommendations elsewhere [28]. Here, neighborhoods with sidewalks on most streets were also associated with meeting 150 min per week or more of moderate-vigorous-physical activity [29]. In another study, lack of sidewalks was inversely associated with walking for leisure [30]. A possible explanation for these inconsistencies is that in some cities, sidewalks may serve more as a barrier than they do as a facilitator for walking. Sidewalks can be of poor quality and badly maintained and when combined with overcrowding, a person’s ability to use them and the enjoyment of doing so is reduced [30].

Our participants who indicated seeing pleasant scenery (interesting things) while walking were more likely to reach 150 min per week or more of transport related physical activity, similar to the results in another study [19]. This implies that the good quality aesthetics in the neighborhood environment may positively influence the transport-related physical activity.

This study also noted that high volumes of traffic along the streets was associated with a lower likelihood of leisure-time physical activity and walking for leisure, which is similar to results found by studies in high-income countries [31]. For example, in the USA, neighborhoods that are safe from traffic were positively associated with walking [32]. Our results suggest that heavy traffic may be a barrier to physical activity and give preliminary evidence of the need to provide safe traffic environments to support physical activity in Africa.

Concerning crosswalks, this study observed that individuals who agreed that the crosswalks in their neighborhood helped walkers feeling safe crossing busy streets were also more likely to report sufficient levels of transported-related physical activity. Again, these results are consistent with those found in other studies [33]. Hence, having crosswalks in neighborhoods with high traffic volume may play an important role in determining the safety and physical activity levels of residents. The results of this study add to the existing, comparable literature by demonstrating that the association between crosswalks and physical activity meets public health recommendations for physical activity in urban and rural (African) settings.

Well-lit streets at night were positively associated with walking for leisure. These findings are significant in a South African context where crime rates are considered to be very high, and increasing with rapid urbanization. A study in the US found that feeling safe was linked to leisure time [33]. Similar results were reported in England [34] and Nigeria [11]. These pointedly demonstrate the need to assess perceived neighborhood attributes and their influence on physical activity [35]. However, limited information in the African context makes direct comparisons with other studies challenging. Perceived safety during the day is related to walking as most of individuals walk for transportation, especially among working class [11]. In addition, this relationship suggests that street lights could act as an indirect indicator for personal safety which in turn promotes walking for leisure as a choice rather than a need.

### Limitations and strengths of the study

Our study has some limitations. It relies on self-reported physical activity and perceived environment, rather than objectively measured physical activity and perceived built environment. Recall bias and imprecise assessment of physical activity could dilute some of the observed associations. In addition, our study is also affected by common sources bias between two self-reported measures which inflate the magnitude of associations. Furthermore, due to the non-availability of cluster-level data, we were unable to account for the clustering effect in the analysis. This has the undesirable effect of generating too conservative standard error, and increasing the risk of type 1 errors. Strengths of this study include the use of both NEWS and IPAQ, which makes it comparable with other studies, globally. Furthermore, this study included a sample population from urban and rural areas that has geographical variability in a perceived built environment.

## Conclusion

We found perceived built environment attributes to be associated with health related physical activity. Our findings provide baseline evidence for the need to provide walkable environments that will make it easier for South African adults to meet physical activity guidelines.

## Abbreviation

IPAQ: International physical activity questionnaire
LTPA: Leisure time physical activity
NEWS: Neighborhood environmental walkability scale
PURE: Prospective Urban and Rural Epidemiology
TPA: Total physical activity
TRPA: Transport related physical activity
WL: Walking for leisure
